# Remimazolam enabled safe anesthetic management during tracheostomy in a patient with amyotrophic lateral sclerosis: a case report

**DOI:** 10.1186/s40981-022-00514-7

**Published:** 2022-03-26

**Authors:** Noriaki Nishihara, Shunsuke Tachibana, Mariko Ikeshima, Ayumi Ino, Michiaki Yamakage

**Affiliations:** Department of Anesthesiology, Sapporo Medical University School of Medicine, South 1, West 16, Chuo-ku, Sapporo, Hokkaido 060-8543 Japan

**Keywords:** Amyotrophic lateral sclerosis, Remimazolam, Flumazenil

## Abstract

**Background:**

Amyotrophic lateral sclerosis (ALS) is known to cause generalized muscle atrophy and respiratory complications. Anesthetic agents and methods for patients with ALS are extremely important because they critically influence postoperative outcomes. In this clinical case, we mainly used remimazolam for safe anesthesia management.

**Case presentation:**

A 66-year-old man had a gradual onset of numbness and weakness in his extremities over 2 years. He was diagnosed with ALS after the appearance of dysarthria and restrictive ventilation disorder. Due to the rapid progression of respiratory dysfunction, the patient was placed on artificial respiration, and a tracheostomy was planned. General anesthesia was induced with remimazolam (6 mg/kg/h) and remifentanil (0.5 μg/kg/min). Tracheal intubation was performed without muscle relaxants, followed by total intravenous anesthesia (TIVA) with continuous administration of remimazolam 0.8–1.2 mg/kg/h and remifentanil 0.3–0.5 μg/kg/min. At the end of the surgery, the anesthetic effect of remimazolam was reversed with 0.4 mg of flumazenil. The patient was discharged from the operating room with stable breathing, and changes to preoperative ventilator settings were not necessary.

**Conclusions:**

We safely performed tracheostomy for a patient with ALS using remimazolam during general anesthesia.

## Background

Amyotrophic lateral sclerosis (ALS) is a progressive neurodegenerative disease that affects both the upper and lower motor neurons in the brain and spinal cord [1]. Typically, ALS initially manifests as weakness of the extremities. However, bulbar palsy is present initially in approximately one-third of cases [2]. ALS causes generalized muscle atrophy and respiratory complications associated with respiratory muscle weakness. Sato et al. reported a case in which apnea and hypoventilation occurred after percutaneous endoscopic gastrostomy (PEG) tube placement and conventional esophagogastroduodenoscopy (EGD) under monitored anesthesia care [3].

Although respiratory complications make anesthetic management more difficult, there are few literature reviews on anesthesia in patients with ALS. An appropriate anesthetic agent and method is extremely important because the selection greatly affects the postoperative outcomes of patients with ALS. In general, the use of rapid reversible short-acting analgesics and sedative agents is recommended [4]. In this clinical case, we mainly used remimazolam during general anesthesia. Together with safe anesthesia management, our patient achieved a stable postoperative respiratory outcome.

## Case presentation

Written informed consent was obtained from the patient for the publication of this case report. A 66-year-old man (height: 160 cm; weight: 46.8 kg; BMI: 18.3 kg/m^2^) experienced a gradual onset of numbness and weakness in his extremities in the past 2 years. The patient was diagnosed with ALS after experiencing dysarthria and restrictive ventilation disorder. In the pulmonary function test, vital capacity (VC) was 23.4% and functional vital capacity was 18.0%. Although initial oxygen saturation (SpO_2_) was 92%, owing to the rapid progression of respiratory dysfunction, SpO_2_ was decreased to 88% and noninvasive positive pressure ventilation (NPPV) was initiated. SpO_2_ was improved to 94%; however, NPPV could not ensure sufficient ventilation and a stable respiratory status. Tracheostomy was anticipated after prolonged artificial respiration. To prevent the exacerbation of respiratory failure and aspiration during surgery, we performed the tracheostomy under general anesthesia.

General anesthesia was induced with remimazolam at 6 mg/kg/h and remifentanil at 0.5 μg/kg/min under monitoring of bispectral index (BIS) with a sensor placed on the bilateral forehead. The BIS value before the induction of anesthesia was 93. Although tracheal intubation was performed without muscle relaxants, gag reflex was not occurred. For anesthesia maintenance, total intravenous anesthesia (TIVA) with continuous administration of remimazolam 0.8–1.2 mg/kg/h and remifentanil 0.3–0.5 μg/kg/min was employed for maintaining a target BIS of 40–60 during surgery. However, because the BIS sensor was close to the surgical site, surrounding noises made accurate measurement difficult. Therefore, to maintain the depth of general anesthesia, the remimazolam dosage could not be reduced. At the end of the surgery, the effect of remimazolam was reversed with flumazenil 0.2 mg as per the attached document. However, this did not awaken the patient and the BIS value was 76. Therefore, we administered an additional 0.2 mg of flumazenil. After using an additional dose, the patient woke up and was discharged from the operating room with stable breathing, and any changes to the preoperative ventilator settings were not necessary. The BIS value after the second administration of flumazenil was 94, which was not different from that before the induction of anesthesia. During anesthesia, gag reflex was not occurred and hemodynamics was stable. The surgery time was 58 min, and the total anesthesia time was 135 min. The patient was discharged 32 days later.

## Discussion

We present a patient with ALS who underwent general anesthesia. When administering general anesthesia to patients with ALS, two important points should be considered. First, from the viewpoint of postoperative respiratory complications, the use of rapid reversible short-acting analgesic and sedative agents is recommended. Currently, most of the analgesic and sedative agents used are short-acting drugs. However, only a limited number of these can be reversed. In view of sedation, desflurane is known to restore pharyngeal function quickly. However, the excretion of inhaled anesthetics depends on respiration, which was unsettling for this ALS patient who was supposed to go back to the general ward. As most ALS patients have respiratory failure, the residual effects of inhalational agents may cause delayed emergence and aspiration. Therefore, we decided to choose TIVA. In the past, propofol was considered ideal for patients with ALS [4]. However, the use of propofol is not necessarily the only choice for patients with ALS in whom residual anesthetic effects are undesirable. Remimazolam can also be reversed. Remimazolam was used for our patient and he returned to the ward safely following general anesthesia.

Second, muscle relaxants should be avoided, although some reports have indicated that the use of muscle relaxants is feasible with careful monitoring, such as train-of-four acceleromyography (TOF-Watch SX, Organon, Dublin, Ireland) [3, 4]. In addition to monitoring, sugammadex can be used to reverse the muscle relaxant effect immediately and safely [5]. In contrast, acceleromyography and sugammadex were reportedly ineffective in another report [6]. In upper motor neuron disease, the reliability of acceleromyography is insufficient because muscle and nerve conditions vary depending on the ALS progression. Similarly, sugammadex may not be effective or the dosage insufficient, resulting in postoperative respiratory complications. In our case, tracheal intubation was performed without using a muscle relaxant.

There were some limitations to the use of remimazolam in our patient. To manage general anesthesia with TIVA, we needed to maintain the depth of anesthesia using a BIS monitor. However, we know that there is a correlation between remimazolam dosage and a decrease in BIS [7]. In our case, the appropriate BIS value was not clear. Moreover, since tracheostomy involves neck surgery, electrical noises interfered with BIS monitoring during the surgery. This may have contributed to the difficulty in measuring the BIS accurately. As a result, excessive remimazolam was administered. Our patient did not emerge from anesthesia following 0.2 mg of flumazenil and an additional 0.2 mg was required. As we previously reported, remimazolam reversal may be beneficial for the anesthetic management of patients with myotonic dystrophy [8]. These cases suggest that the use of remimazolam in neuromuscular diseases is superior because it can be reversed. To reverse remimazolam and establish safe anesthetic management, more effective neuromonitoring devices are needed.

## Conclusion

We safely managed general anesthesia using remimazolam during tracheostomy for a patient with ALS. Remimazolam may be beneficial in the anesthesia management of patients with neuromuscular diseases because of its reversibility.

## Data Availability

Not applicable due to patient privacy concerns.

## Abbreviations

ALS: Amyotrophic lateral sclerosis
TIVA: Total intravenous anesthesia
NPPV: Noninvasive positive pressure ventilation
BIS: Bispectral index
